# Post-introduction evaluation (PIE) of rotavirus vaccine in India

**DOI:** 10.1016/j.jvacx.2024.100526

**Published:** 2024-07-17

**Authors:** Pawan Kumar, Arindam Ray, Amrita Kumari, Amanjot Kaur, Rhythm Hora, Kapil Singh, Rashmi Mehra, Seema S Koshal, Shipra Verma, Syed F. Quadri, Arup Deb Roy

**Affiliations:** a Immunization Division, Ministry of Health and Family Welfare, Government of India, India; b Bill and Melinda Gates Foundation, Delhi, India; c John Snow India Pvt Ltd, Delhi, India

**Keywords:** Immunization, Rotavirus vaccine, Post introduction evaluation

## Abstract

**Background:**

India became the first country in the WHO South-East Asia Region (SEAR) to introduce the rotavirus vaccine (RVV) in the Universal immunization programme (UIP) in 2016 with nationwide expansion by 2019. It was a landmark move to reduce the diarrheal disease burden in under-five children. To assess the implementation process of introduction of RVV, Post Introduction Evaluation (PIE) was conducted in March 2022.

**Methods:**

The evaluation was conducted across 14 states, 28 districts and 28 health facilities to obtain a nationwide geographical inclusion. Stakeholders involved in program decision-making, planning, training, vaccine delivery, logistics, and communication from all levels (National, state, district, health facility, health worker, caregiver) were interviewed using standardized data collection tool for PIE (adapted from the standard WHO PIE questionnaire) and scripted on a digital tool.

**Results:**

A total of 260 interviews were conducted. Political willingness, well-planned preparedness activities, securing vaccines timely, strong supply chain monitoring, availability of domestic RVV products, quality trainings and intense communication activities were the key factors identified for the successful RVV introduction. Key activities during the introduction included cold chain space assessment, trainings of healthcare workforce, dissemination of job aids, updation of recording & reporting formats and strengthening of AEFI surveillance. Lack of community awareness for immunization in a few areas, fear of AEFI amongst some caregivers and local issues with Alternate Vaccine Delivery (AVD) were some reported challenges in achieving high coverage for RVV.

**Conclusions:**

Overall, the nationwide roll-out of RVV was smooth and the vaccine has been well-accepted in the community. The assessment emphasizes on having a well-strategized operational and communication planning, which is very crucial for any new vaccine introduction.

## Highlights

• Rotavirus vaccine (RVV) post-introduction evaluation (PIE) was conducted in March 2022 to assess the implementation process of RVV introduction in India.
• One of the largest PIE conducted in terms of geographical scope (14 states, 28 districts, 28 health facility).
• First PIE that used a digital tool for data collection at all the levels.

## Introduction

**Fig. 1:**
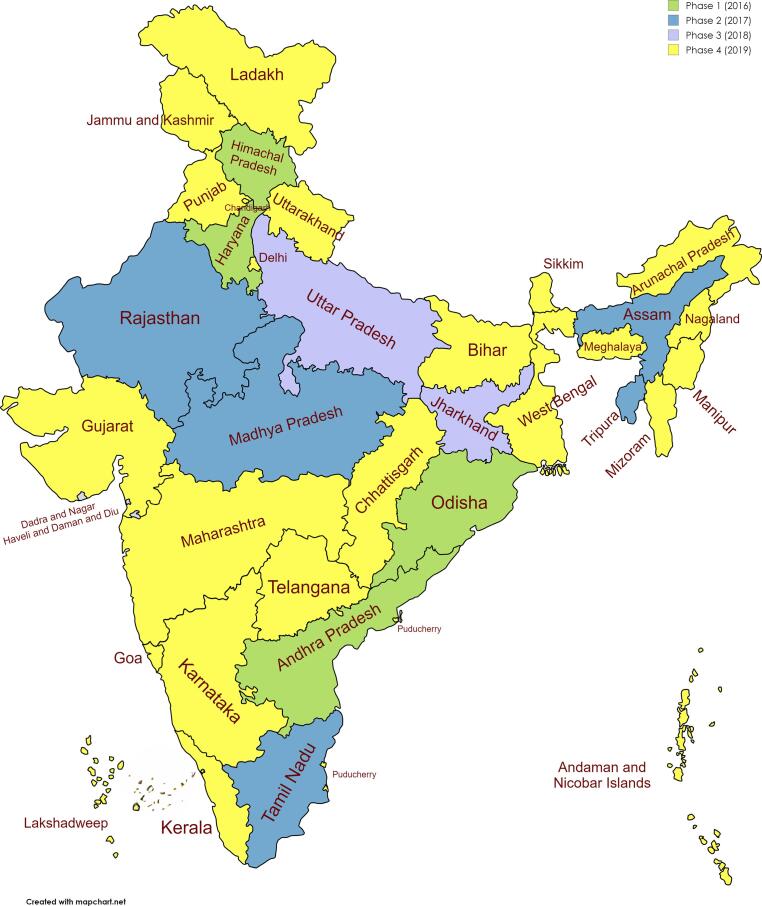
Phased roll-out of RVV from 2016 to 2019.

Rotavirus gastroenteritis is a major cause of hospitalization in children <5 years in India [1]. Approximately 40% of children under 5 years of age hospitalized for acute gastroenteritis had rotavirus disease during 2005–2009 [2]. As per 2011 estimates, rotavirus is responsible for more than 11 million diarrhea episodes, 3.27 million OPD visits, 0.87 million hospitalizations & 0.07 deaths in children every year in India in the first 2 years of life [3]. Rotavirus vaccine (RVV) has been shown to be effective in reducing diarrhea due to rotavirus, with greater effectiveness against moderate and severe diarrhea [4]. In March 2016, India became the first country in the World Health Organization South East Asia Region (WHO SEAR) to introduce RVV in the Universal Immunization Program (UIP). Under the national immunization schedule, RVV is given in a 3-dose schedule at 6 weeks, 10 weeks, and 14 weeks, along with other vaccines under the UIP[5]. Between 2016 and 2018, 11 states (Andhra Pradesh, Haryana, Himachal Pradesh, Jharkhand, Odisha, Assam, Tripura, Rajasthan, Tamil Nadu, Madhya Pradesh, and Uttar Pradesh) introduced RVV in a phased manner, covering 56.4% of India’s birth cohort. In 2019, RVV was expanded to the remaining 25 states and union territories under the ‘100 days agenda’ of the Government of India covering the entire country by September 2019. Fig. 1 depicts the phased roll-out of RVV from 2016 to 2019.

WHO recommends conducting a post-introduction evaluation 6–12 months following a new vaccine introduction to highlight best practices, identify challenges, and implement timely program improvements [6], [7]. Following the RVV vaccine introduction in the country, JSI India, in collaboration with the Ministry of Health and Family Welfare and development partners, conducted a post-introduction evaluation in March 2022, with the primary objective of assessing the implementation process and evaluating the overall impact of introduction of a new vaccine focusing on related programmatic issues and its community receptiveness. The evaluation aimed to assess the implementation process and evaluate the overall impact of introduction of a new vaccine focusing on related programmatic issues and its community receptiveness.

## Methods

The present study is a mixed method study comprising of qualitative and quantitative components conducted across selected states and districts in the country. The data was collected under the following parameters: decision-making, planning, and implementation of RVV introduction through the post-introduction evaluation.

*Sampling:* As per the WHO recommendation, post-introduction evaluation (PIE) should be conducted in at least six regions (or provinces) to provide adequate information on the new vaccine introduction under consideration. However, given the diversity of the country, phase-wise introduction, usage of different types of RVV products under UIP, and recent transitioning of the RVV products, it was decided to expand the sample size and select 14 provinces/states. All 36 states and union territories (UTs) of the country were categorized into 6 zones comprising North, South, East, West, Central, and North-East and at least one state from each zone was selected. At least one state was selected among the 4 phases of the introduction of RVV. Considering the various RVV products, 9 states where liquid frozen RVV was initially introduced and 5 states where lyophilized RVV was initially introduced were selected. It was also ensured that the 14 selected states also include states where RVV product transition is ongoing/planned.

The district selection was based on programmatic parameters, including the inclusion of the district in Intensified Mission Indradhanush (IMI 4.0). IMI is a catch-up campaign conducted to increase the full immunization coverage of children. The IMI districts are based on immunization coverage, VPD surveillance, and demographic data. Therefore, any district being chosen for IMI was considered to be low performing. On this basis, in each RVV PIE state, 1 IMI and 1 non-IMI district were selected to cover the experience of RVV introduction in both well performing and underperforming districts. Once the districts were grouped into IMI and non-IMI districts, the final selection was carried out using a convenient sampling method considering two other parameters; vaccine availability index 2021–22 and the female literacy rate (data taken from NFHS-5). Table 1 depicts the selection of the states and districts based on the above inclusion criteria.

**Table 1:** States and districts selected for the RVV PIE.

Sr.No.	State/UTs	Geographical Zone	Districts		RVV product Rotavac®/Rotasiil® lyophilized	Phase of RVV introduction	Transitioning of products (Rotasiil® Lyophilized/Rotavac® to Rotasiil® liquid
			**IMI**	**Non-IMI**			
1.	Assam	North-East	Tinsukia	Kamrup (Metro)	Rotavac®	Phase-2	__
2.	Bihar	East	Patna	Jehanabad	Rotavac®	Phase-4	__
3.	Delhi	North	East district	South-west district	Rotavac®	Phase-4	__
4.	Gujarat	West	Kheda	Ahmedabad	Rotasiil®	Phase-4	Rotasiil® Lyophilized to liquid
5.	Karnataka	South	Bengaluru (Urban)	Dakshina Kannada	Rotasiil®	Phase-4	Rotasiil® Lyophilized to liquid
6.	Kerala	South	Thiruvananthapuram	Kottayam	Rotasiil®	Phase-4	Rotasiil® Lyophilized to liquid
7.	Madhya Pradesh	Central	Jabalpur	Indore	Rotavac®	Phase-2	__
8.	Maharashtra	West	Pune	Nagpur	Rotasiil®	Phase-4	Rotasiil® Lyophilized to liquid
9.	Odisha	East	Cuttack	Puri	Rotavac®	Phase-1	Rotavac® to Rotasiil® liquid
10.	Punjab	North	Faridkot	SAS Nagar	Rotavac®	Phase-4	__
11.	Rajasthan	West	Dausa	Pali	Rotavac®	Phase-2	__
12.	Tamil Nadu	South	Thiruvallur	Vellore	Rotavac®	Phase-2	Rotavac® to Rotasiil® liquid
13.	Uttar Pradesh	North	Lucknow	Baghpat	Rotavac®	Phase-3	__
14.	West Bengal	East	North 24 Parganas	Kalimpong	Rotasiil®	Phase-4	Rotasiil® Lyophilized to liquid

Within each district, based on operational feasibility, one health facility and in the catchment area of the selected facility, one sub centre was selected. Thus, a total of 14 states, 28 districts, 28 health facilities, and 28 health sub-centers were identified for the RVV PIE.

***Data collection*:** Data collection consisted of three primary components: (1) desk review of planning documents, (2) interviews with key stakeholders (health officials, health care providers, and beneficiaries), and (3) on-site observation at session sites and cold chain stores.

We used the standard WHO PIE tool, modified and conceptualized for RVV PIE in the Indian context. A mix of quantitative and qualitative approaches were employed for data collection. The main thematic areas under which the data was collected comprised of introduction planning and coordination, training, vaccine delivery and coverage, data recording and reporting, cold chain and vaccine logistics, monitoring, waste management, injection safety, advocacy, communication, and attitudes and perception among caregivers. Apart from these, safety surveillance component was also looked into.

The process of data collection, synthesis, and analysis was digitized, and the questionnaires developed for all the various levels were scripted on a web-based digital tool. The survey tool developed was compatible with smartphones, tablets, and laptops and allowed evaluators to save drafts, complete surveys in the offline mode and submit once they are in an area with internet access.

The tool was pilot-tested in the field before finalization. A one day national-level orientation was conducted on 21st Mar’22 for the RVV PIE where 64 evaluators from 16 partner organizations were briefed on tools and other aspects where hands-on training to install the tool and fill in the data were ensured. The RVV PIE tool was hosted on a secure IT platform to ensure data security. For each district, a separate user-id and login password was issued to maintain data confidentiality. The data collection was conducted from 23rd-26th Mar’22.

## Results

A total of 260 interviews at different levels and community areas were conducted. Synthesized findings on RVV program planning, and implementation across each main area are discussed below.

### Decision-making, introduction planning, and coordination

Following the recommendation of the National Technical Advisory Group on Immunization (NTAGI) and after approval of Mission Steering Group (MSG), states were identified for phase-wise RVV introduction based on criterions such as diarrhoeal disease burden, AEFI preparedness, RI coverage & system preparedness. For the smooth introduction of RVV in the country, various preparatory activities were conducted including preparedness assessment, development of training and communication packages, cascade training for programme managers and health workers, strengthening of AEFI system, and supply chain strengthening. Based on the discussion with the National-level stakeholders during PIE, it was evident that several policy level decisions and innovations were undertaken at national level for smooth introduction and implementation of RVV. Notable amongst these are the usage of Rotavac® without the buffer that mitigated various programmatic challenges [8] such as difficulty in delivering high volumes through oral route, confusion amongst health workers for a sequence of buffer and vaccine administration leading to errors, sparking anxiety for the child before actual vaccine administration, etc. The other policy decision made in the program was following the uniform cold chain storage and transport policy in-line with the Vaccine Vial Monitors (VVMs) applicable for oral poliovirus vaccine (OPV) as both Rotasiil® and Rotavac® do not follow open vial policy and cannot be used after 4 h of reconstitution/opening of the vial. Therefore, it was ensured that the VVM of RVV was placed on the cap of the vial and not on the body of the vial. Thirdly, the RVV dropper size and color were modified to avoid confusion with the OPV droppers. A similar modification was made in the oral syringes for the delivery of the Rotasiil® vaccine so that it is not used for the delivery of other UIP vaccines through injection. Also, the size of the adapter supplied with Rotasiil® was such that no other syringe available in the programme/in the market will fit into the adapter supplied.

### Programme operations and management

RVV PIE tried to capture the operational aspects of RVV introduction in UIP. The key steps that were taken prior to the introduction of RVV are summarized in Table 2.

**Table 2:** Key steps taken prior to the introduction of RVV.

Key steps	State	District	Health Facility
Additional training of Cold Chain Handlers (CCHs)	85.7 %	92.9 %	84.6 %
Job aids for Cold Chain Handlers (CCHs) and vaccinators	100 %	75 %	76.9 %
Cold chain space assessment	85.7 %	85.7 %	73.1 %
Cold chain space augmentation (wherever required)	28.6 %	35.7 %	_
Update manual recording and reporting formats	78.6 %	60.7 %	73.1 %

Job aids for CCHs and vaccinators were reported to be distributed at all the states. Out of these, the same was available at 75% of the districts and 77% of the health facility level surveyed. More than 78% of respondents at the state level, 61% at the district level, and 73% at the health facility level reported that manual recording & reporting formats were updated. The e-VIN system was also updated to enable entries for RVV stock prior to the introduction of the vaccine. It was found that all the states carried out preparedness assessment. Around 50% of respondents at the state level and 68% at the district level reported that RI microplans were updated prior to the RVV introduction.

### Capacity building/training

Training of the health workforce (program managers, medical officers, data handlers, cold chain handlers, supervisors, vaccinators, and social mobilizers) is crucial for any new vaccine introduction in the routine immunization programme. In all the surveyed states, the cascade model of training was followed for RVV introduction and was supported by the National Health Mission (NHM) funds. The training was done using the operational guidelines, FAQs and leaflets which were also shared with the participants [9]. At the district level, operational guidelines for RVV introduction were provided in 96.4% training (except Kalimpong, West Bengal, as it was part of another district at the time of RVV introduction), leaflets were provided in 92.9% of the training (except SAS Nagar, Punjab and Cuttack, Orissa). One-page information on steps in administration of Rotasiil® vaccine was distributed in all Rotasiil® states. All immunization partners provided technical support in conducting the training. JSI India, as the lead techno-managerial partner, supported the regional/state-level training. The training was also monitored by the Government representatives and, partners and nearly two third of states (64.3%) surveyed used a standard checklist to monitor the training. Pre and post-tests were conducted to assess the quality of trainings. In the training for new vaccine introduction, station approach was used extensively up to the district level by dividing the participants in small groups to break the monotony of classroom training & for better clarity as a novel approach to teaching. In all the participating states, the trainings were completed prior to vaccine introduction. Nearly three-quarters (72%) of the evaluated districts completed training at least one month before RVV introduction. The remaining districts completed training at least 15 days prior to the launch of RVV. Nearly 73.3% of the respondents appreciated hands-on training & 70% of them said that the extensive use of FAQs during the training helped them to understand the key points easily.

Nearly two-thirds (64.3%) of the health workers responded that they had attended training on micro plan revision within the last 1 year, 3.5% within 1–2 years, while 17.9% of them attended such training more than 2 years ago & 14.3% of them were unable to recall.

### Immunization supply chain

Prior to the introduction of RVV, multilevel cold chain space assessment was conducted at the state, district & health facility level. Accordingly, cold chain space augmentation was done (repair of existing equipment, rationalization of available equipment, and installation of new equipment) in nearly 30% of the states and 36% of districts participating in the study. Evaluators visited 28 cold chain points at state, district, and health facilities. It was observed that states & districts didn’t usually rely on a single system to monitor the temperature of the cold chain equipment at cold chain points (CCPs). Around 64.3% and 60.7% of respondents at the state and district level, respectively, stated that both alcohol thermometers, as well as electronic-vaccine intelligence network (e-VIN) sensors were used for temperature monitoring. States and districts where e-VIN temperature loggers were not functional/not installed (28.6% states & 39.3% districts surveyed) rely solely on alcohol thermometers for temperature monitoring. Review & monitoring mechanism for the immunization supply chain (ISC) at various levels was reported to be mainly through eVIN & field visits by VCCM. It was observed that 93% of the states, all the districts & 88.5% of health facilities use the eVIN for review and monitoring. Amongst those surveyed, 85.7% of states, 75% of districts and 46.2% of health facilities also use the monitoring field report of VCCMs for review of the ISC. Periodic field assessments are carried out for review and monitoring in more than 57% of the district and health facilities surveyed. Before the introduction every cold chain point was assessed for space availability for the new vaccine. During the PIE, it was observed that the record-keeping for vaccine wastage is variable, and there is sub-optimal monitoring of this activity. At the state, district, and health facility level, 64.3%, 53.6%, and 60% of respondents, respectively, stated that multi-dose vial is the major cause of vaccine wastage. The other major cause reported for high wastage was vaccines with a ‘no open vial’ policy (50–52% respondents at state, district, and health facility level). At the state level, all the respondents said that vaccine waste disposal has been outsourced, and at the district level nearly 90% stated that waste disposal was outsourced. In 10% of the districts, safety pits were used for vaccine waste disposal.

### Communication

Communication activities play a very important role in any new vaccine introduction to create visibility, generate demand, and build confidence in the community in immunization programmes, leading to improved coverage. Prior to the launch of RVV, ceremonial launch, and media sensitization were carried out in all states. Media sensitization workshops were held in 71.4% of the districts. The most common strategy adopted at the state and facility level to inform the community about RVV, its benefits, schedule, eligible beneficiaries was the use of posters while at the district level, interpersonal communication by FLWs, posters, advocacy with key stakeholders and use of social media. Approximately 50% of the respondents at the state and district level also reported placing RVV within the overall diarrhea control program, and messaging during Intensified Diarrohoea Control fortnight (IDCF) that consists of a set of activities to be implemented in an intensified manner once in a year for 14 days for prevention and control of deaths due to dehydration from diarrhea across all the States & UTs. Other innovative practices for communication reported by the respondents at the district level included LED vans, catchy radio jingles, influencer & Non-Government Organization (NGO) mapping, balloon IEC (information, education, communication), wall paintings, vaccination slip (Tika nimantran patrika), appreciation certificate, beneficiaries as the brand ambassador, area-specific IEC in regional languages. Another important strategy to inform the community is interpersonal communication by frontline health workers. At the facility level, it was found that IPC activities were undertaken by 80% of the health facilities surveyed. At the health worker level, the main strategies for communication of RVV introduction were discussion during ASHA/AWW meetings (93.5%) and meeting with mothers/family members (83.9%). Key channels used to inform the community about RVV introduction at the district level and facility level were Newspaper, Television, social media (WhatsApp, Facebook, and Twitter), Community groups and Radio.

### Supervision, monitoring and review

A new vaccine introduction provides an opportunity for health system strengthening as vaccines are introduced within the ambit of routine immunization (RI) programme. It enables convergence with the line departments and partners through the State Task Force on Immunization and District Task Force on Immunization meetings which are held in the presence of administrative heads of the district and state, other government officials and immunization partners. As per norms, one STFI meeting is to be organized every quarter and one DTFI meeting is to be conducted every month. It was found that STFI and DTFI were done at the same platform as for the review of COVID-19 vaccination campaign and pandemic control activities. However, how extensively routine immunization was discussed during COVID-19 meetings was not clear. Hence in all the surveyed states and districts, the norms for STFI and DTFI were easily met.

During the supportive supervision and monitoring field visits, the key issues identified by the evaluators include the need for better supervision, non-availability of updated micro plan, vaccine hesitancy, mismatch of vaccine stock data with physical stock available, need for refresher training, and poor understanding of the concept of the interchangeability of RVV products (Table 3). The review meetings at the block, district, and state levels are the major forums in which these issues are discussed for corrective actions.

**Table 3:** Key observations of supportive supervision from the field.

Sr. No	Observation in field	% of States (N=14)	% of Districts among the reported states(N=28)
1	Need for better supervision	71.4	42.9
2	Updated microplans not available	42.9	53.6
3	Vaccine hesitancy	35.7	35.7
4	Mismatch of vaccine stock data with physical stock	28.6	14.3
5	Need-based catch-up and refresher training	21.4	14.3
6	The concept of interchangeability not understood	21.4	28.6

The state level respondents informed that monitoring observations were discussed in the review meetings and feedback letters were sent to concerned districts as needed. Some states stated that there was no fixed mechanism to address the field issues, and they are being handled on a case-to-case basis depending on the requirement. At the district level, in addition to discussion in review meetings and feedback letters, some districts also sought support from immunization partners and line departments to address issues identified. The feedback is given to the health workers during supervisory visits. The health workers surveyed reported that the key feedback provided to them during supervisory visits included errors in recording and reporting (35.7% of respondents), poor mobilization of beneficiaries and lack of availability of RCH registers (10.7% each), poor identification of eligible beneficiaries, MCP cards not available, incorrect technique of vaccine administration and due list not available (7.1% each). There are certain state-level innovations done to strengthen monitoring and supervision activities such as categorizing the blocks and health sub-centers into different categories (A, B, and C with A being the best and C being the poorest) based on a set of key programmatic indicators (Bihar). In Tamil Nadu, the health supervisors and District Immunization Officers monitor the same area (same village site/block) for 3 consecutive months to identify any gaps.

### Data management

Majority of the surveyed states use HMIS data for monitoring coverage of RVV and other vaccines. Other data sources such as concurrent monitoring data, RCH portal, state’s own portal, evaluation data are also used for monitoring vaccine coverage, at state and district level. Six states reported that they use their own portal for coverage monitoring that has name-based reporting in addition to HMIS: West Bengal-Matrima portal, Gujarat-Technology Enabled Community Health Operations (TeCHO+), Tamil Nadu-Pregnancy & Infant Cohort monitoring & Evaluation (PICME), Rajasthan-Pregnancy, Child Tracking & Health Services Management System (PCTS), Assam-Assam Progressive Routine Immunization Performance Tool (APRIP), Delhi-State MIS (Management Information System) portal for monitoring of coverage. However, multiple data sources result in discrepancies between the state and the HMIS data reporting.

### Vaccine management, cold-chain, transport, injection safety, and waste management

In total, the evaluation team observed 28 cold chain points and dry storage areas. All cold chain storage units were noted to be clean and functioning, including internal thermometers and good cold chain practices were observed at all sites visited. Syringes for reconstitution and administration of RVV product (Rotasiil) were stored optimally in a clean, dry, and organized manner at all cold chain points. The evaluation team did not observe any expired or unusable vaccine, and none was reported. Vaccine fridge temperatures were recorded twice daily in temperature log books, including weekends and holidays. Job aids for vaccine management and storage were visibly displayed at all cold chain points.

### RVV products in UIP

It is pertinent to note that different RVV products are being used under the UIP. The PIE explored the challenges in product switch (based on the interchangeability guidelines shared by MoHFW) and the use of multiple RVV products in the programme. It was found that in all the districts/health facilities surveyed, training has been provided to all the health personnel for the product switch. To guide the health care workers and cold chain handlers in the field for the RVV product switch, one-pager job aids were developed and shared with the states by MoHFW. However, the distribution of the same further within the states was reported to be sub-optimal (only 50% of the districts). At the health facility level, only 40% of the districts reported having received job aid from the districts. Overall, the respondents in these 7 states reported that the product switch activities were more or less smooth. However, as observed in the RVV PIE, only 71.4% of health workers interviewed were aware that RVV products are interchangeable. During the transition from one type of RVV to another, tracking of the vaccine stock availability was done by eVIN and declarations from the block on stock of old RVV products.

Major challenges reported for Rotasiil® lyophilized were reconstitution, additional training, operational inconvenience, time-consuming process. At health worker level the most common challenges faced were long reconstitution time and spitting of vaccine by the child due to large volume.

### Session site

Evaluators observed that most of the health workers (89.7%) handled vaccines correctly at the session sites. The open vial policy was properly implemented & immunization safety guidelines for RI were followed at all session sites. It was reported that neither any expired vaccine nor any vial with VVM at the unusable stage was supplied to the session sites in the last 6 months. As observed at the session sites, 80% of health care workers provided message on the name of vaccine(s) and disease(s) prevented by the vaccine(s) administered to the child, 77% informed the mother when to return, 73% advised mothers to bring the vaccination card during the next visit and 67% informed about possible adverse events. No instances were recorded where Auxiliary Nurse Midwife (ANM) was not sharing any key messages with the mothers/caregivers. Overall, 53% of the ANMs observed conveyed all 4 key messages to the mothers after vaccination at the session site. It was found that 80% of the caregivers were aware about the RVV introduction in immunization programme. Most of the caregivers (98.2%) reported that child had no adverse event after administration of RVV.

When caregivers (56) were asked about their views on new vaccine introduction, twenty-six stated that new vaccines in RI have increased overall acceptance of vaccine. Amongst them, 18 stated that vaccines are beneficial for their children, 15 respondents said they prevent some diseases and 2 respondents stated that more new vaccines should be introduced in UIP.

### Safety surveillance

Monitoring of adverse events following immunization (AEFI) is an essential strategy for ensuring the safety of vaccines that provides valuable information to help plan and take necessary actions to sustain public confidence in vaccine safety. Safety surveillance training is a component of overall training for new vaccine introduction. Surveillance and immunization officers at state and district level did not report cases of intussusception among infants as AEFI in the previous year. As vaccine safety is an essential component guiding its smooth implementation on the ground, WHO has recommended monitoring intussusception cases for the recent rotavirus vaccines [10]. None of the state and district surveillance officers were aware of any additional surveillance for intussusceptions.

## Discussion

Post Introduction Evaluation for Rotavirus vaccine provided an excellent opportunity to understand its introduction under UIP. Pre-introduction efforts including preparedness assessment, training, political willingness and support from State Governments & immunization partners were reported to be the key enablers for introduction of RVV.

Prior to introduction of the rotavirus vaccine under UIP, preparedness activities such as securing vaccine supplies at national level, IEC to create awareness to facilitate demand generation for new vaccine and training of health personnel were undertaken. Assessment of cold chain space was also done in all States and where required cold chain space was augmented. A study on introduction of rotavirus vaccine under UIP highlighted that assessment of the cold-chain and augmentation before the introduction of RVV as one of the success factor.[11] Moreover, the Programme Implementation Review (PIR) revealed that the RVV introduction had strengthened the supply chain and there were no shortages of the logistics for RVV introduction.[12] Training materials were disseminated and training at all level was completed before the introduction. Strong IEC along with use of digital & social media helped to create positive awareness among beneficiaries. The importance of robust communication systems in guiding the smooth roll-out of the RVV vaccine across all geographies in country was noted in the previous literature as well.[11], [12] Overall, the introduction of vaccine was smooth and acceptance of new vaccine among community has been good. This has been achieved through partner coordination as highlighted in previous studies as well.[13].

Key challenges in achieving high RVV coverage (and possibly other UIP vaccines) by respondents at all levels were shortage of manpower particularly ANM, lack of rationale deployment of ANM, lack of flexibility in the programme to allow rationale adjustments in AVD payments, lack of awareness of the community about immunization and fear of AEFI amongst caregivers. This is in line with the previous literature, that mentions limited service provider availability and no catch-up training for new recruits were some of the barriers to RVV coverage. [14] Infrequent supportive supervision visits, mismatch of vaccine stock data with physical stock, gaps in knowledge of health care workers and vaccine hesitancy amongst community were key observations made during monitoring visits to the field. A need to emphasize on regular monitoring and supportive supervision at all levels has been recommended from previous literature. [14] Lack of knowledge on inter-changeability of RVV vaccine products amongst health workers has been highlighted in the review.

The limitations of the PIE include delays in carrying out PIE due to COVID-19-related restrictions and engagement of health staff in COVID-19-related activities. Also, there is a possibility of recall bias by the respondents from the states where RVV was introduced in an initial phases and language barriers in some districts for evaluators conducting the PIE (largely overcome with the help of accompanying local staff).

## Recommendations

**Fig. 2:**
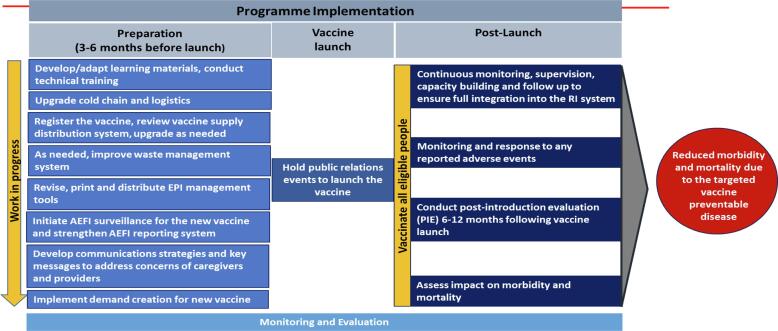
New vaccine introduction strategy.

The introduction of any new vaccine must be planned taking care of all programmatic components. The template given in Fig. 2 can be contextualized for the introduction of a new vaccine in the routine immunization program.

As per the evidence gathered during the PIE, the various measures taken for the successful introduction of RVV should continue for future new vaccine introductions.

• Operational guidelines, training, and IEC materials, including job aids for new vaccine introduction, should be developed and disseminated at all levels. Comprehensive training packages, including user-friendly digital training tools is essential to build the capacity of the FLWs, supervisors and programme managers. Using new-age training tools for training of health personnel can be effectively leveraged.
• Vaccine supplies to be secured by advance procurement, keeping lead time in mind. Having a uniform vaccine product will ensure seamless planning and implementation of a new vaccine in the UIP. Frequent change of vaccine products brings training, supply chain and other programmatic challenges.
• The preparedness for new vaccine introduction should be reviewed regularly at all levels starting 3–4 months before expected date of introduction using various platforms.
• Desk reviews and field visits should be conducted by government and non-government stakeholders to review preparedness. Triangulation of data from various sources such as eVIN, HMIS, and concurrent monitoring to generate logical reason analysis for low coverage.
• Ceremonial launches in state and districts and other media events should be planned to inform the community and all stakeholders, including private practitioners and professional bodies for new vaccine introduction.
• To improve coverage in urban areas, due focus needs to be put on innovative strategies for urban & *peri*-urban areas and migratory population such as clear demarcation and allocation of area amongst health workers, intense IEC to inform community regarding session details etc. Apart from this, special catch-up drives are also needed to improve coverage in these areas.
• Post-introduction monitoring and review to be done at all levels to ensure vaccine uptake.
• Under the guidance of the Immunization Division, clear roles and responsibilities of partner agencies lead to seamless partnerships.

## Conclusion

The selection of 14 states for RVV PIE made it a comprehensive exercise. Overall, the introduction of RVV was smooth, and acceptance of the vaccine in the community has been good. Key underlying factors that led to a successful RVV introduction in the country have been the political willingness, strong leadership, sound preparedness activities, availability of domestic products, support of immunization technical partners, completion of quality training at all levels, use of print & mass media and other communication strategies for demand generation, etc. However, a few challenges, such as sub-optimal awareness in few pockets, fear of AEFI, and local issues with Alternate Vaccine Delivery (AVD) were reported.

Key action points to improve RVV and overall RI coverage include catch up drives like IMI, generating demand for immunization services, updation of microplans to include all catchment areas, strengthening of recording and reporting system and involvement of line departments. Focus needs to be put on urban & *peri*-urban areas and migratory populations with need-based and locally adaptable strategies.

Support from immunization partners (JSI, WHO, UNICEF, UNDP) in capacity building, preparedness assessment, supportive supervision and monitoring, developing communication strategy, strengthening AEFI surveillance and monitoring of supply chain have been instrumental in a successful introduction of the RVV.

This exercise has provided an excellent opportunity to understand RVV introduction under UIP and provide learnings about the areas of improvement in implementation of new vaccines which can be replicated at regional and global level.

## CRediT authorship contribution statement

**Pawan Kumar:** Project administration, Writing – review & editing. **Arindam Ray:** Conceptualization, Supervision. **Amrita Kumari:** Conceptualization, Writing – original draft. **Amanjot Kaur:** Methodology, Visualization. **Rhythm Hora:** Investigation, Methodology. **Kapil Singh:** Formal analysis, Supervision. **Rashmi Mehra:** Formal analysis, Visualization. **Seema S Koshal:** Investigation, Methodology. **Shipra Verma:** Investigation. **Syed F. Quadri:** Resources, Software. **Arup Deb Roy:** Conceptualization, Project administration, Supervision, Writing – review & editing.

## Declaration of competing interest

The authors declare the following financial interests/personal relationships which may be considered as potential competing interests: Amrita Kumari reports writing assistance was provided by Bill & Melinda Gates Foundation.

## Data Availability

Data will be made available on request.
